# The diversity of human papillomavirus infection among human immunodeficiency virus-infected women in Yunnan, China

**DOI:** 10.1186/s12985-014-0202-3

**Published:** 2014-12-07

**Authors:** Hong-Yun Zhang, Man-Dong Fei, Yong Jiang, Qiu-Yue Fei, Hong Qian, Lin Xu, Yu-Ni Jin, Cheng-Qin Jiang, Hai-Xia Li, Sarah M Tiggelaar, Jennifer S Smith, Vikrant V Sahasrabuddhe, You-Lin Qiao

**Affiliations:** Department of Gynecology and Obstetrics, The First Affiliated Hospital of Kunming Medical University, Kunming, Yunnan China; Centre for Disease Control and Prevention of Yunnan Province, Yunnan, China; Cancer Institute & Hospital, Chinese Academy of Medical Sciences & Peking Union Medical College, Beijing, China; Women and Children’s Hospital of Luxi County, Luxi, Yunnan China; Vanderbilt University, Nashville, TN USA; University of North Carolina at Chapel Hill, Chapel Hill, NC USA

**Keywords:** HPV, Genotypes, HIV, Cervix, China

## Abstract

**Background:**

Yunnan has one of the oldest and the most severe human immunodeficiency virus (HIV) epidemics in China. We conducted an observational study to evaluate the human papillomavirus (HPV) genotype distribution in relation to cervical neoplastic disease risk among HIV-infected women in Yunnan.

**Methods:**

We screened 301 HIV-infected non-pregnant women in Mangshi prefecture in Yunnan province. All consenting participants underwent simultaneous and independent assessment by cervical cytology, colposcopy-histopathology, and HPV genotyping. Unadjusted and multivariable-adjusted multinomial logistic regression analysis was conducted to evaluate factors associated with single or multiple carcinogenic HPV genotypes.

**Results:**

HPV genotypes were present in 43.5% (131/301) overall, and carcinogenic HPV genotypes were present in 37.5% (113/301) women. Among women with carcinogenic HPV genotypes, 80 (70.8% of 113) had a single carcinogenic HPV type, while 33 (29.2%) women had multiple (2 or more) carcinogenic HPV types. Overall, the most common carcinogenic HPV types were HPV52 (7.3%), HPV58 (6.6%), HPV18 (6.3%), HPV16 (6.0%), and HPV33 (5.3%). In women with cervical precancerous lesions (i.e., high-grade squamous intraepithelial lesions [HSIL] on cytology or cervical intraepithelial neoplasia grade 2 or worse [CIN2+] detected on colposcopy-histology), the most commonly detected genotypes were HPV16 (28.6%), HPV52 (25.0%), HPV58 (17.9%), HPV18 (10.7%) and HPV31 (10.7%). Increasing age was an independent risk factor associated with presence of single carcinogenic HPV types (adjusted odds ratio: 1.04, 95%CI: 1.01-1.07, *p* = 0.012) but not with the presence of multiple carcinogenic types in the multivariable-adjusted models.

**Conclusions:**

As HIV-infected women continue to live longer on antiretroviral therapy in China, it will be increasingly important to screen for, and prevent, HPV-associated cervical cancer in this population, especially given the wide diversity and multiplicity of HPV genotypes.

## Background

Invasive cervical cancer (ICC) is a leading cause of cancer related morbidity and mortality afflicting women globally, with over 527,000 annual incident cases and over 265,000 annual deaths [1]. An estimated 50,000 new cases are diagnosed and 38,000 women die annually due to ICC in China [1]. Persistent infection with carcinogenic genotypes of the human papillomavirus (HPV) is the necessary etiologic factor for ICC. Of the 200+ identified HPV types, approximately 40 types can infect the human anogenital tract, and HPV16 and 18 are associated with ~70% of ICC cases [2].

Women with immunosuppressive conditions, such as human immunodeficiency virus (HIV) infection/acquired immunodeficiency syndrome (AIDS), have been shown to have a higher prevalence of cervical HPV infection and higher frequency of multiple HPV genotypes than women from the general population [3]. The advent of antiretroviral therapy (ART) for treatment of HIV/AIDS has significantly increased the life-spans of HIV-infected women, yet has increased their risk for a prolonged period of persistence of HPV infection, and consequently increased risk of progression to cervical precancer and cancer [4]. HIV-infected women are 4–5 times more likely to have ICC compared to HIV-uninfected women and ICC has been classified as an AIDS-defining malignancy [5].

By the end of 2011, it was estimated that there were 7.8 million persons living with HIV in China [6]. The overall national HIV prevalence estimates remains low (0.058%), but the epidemic is concentrated in some geographical regions such as Yunnan, a province in Southwestern China with a high HIV burden, accounting for 34.8% of the estimated number of cases nationally [7]. Due to limited availability and awareness of screening for cervical cancer, few women are ever screened. Limited data is available on the burden and genotypes of HPV among HIV-infected women in this or other settings in China [8-10]. To expand the evidence in this area, we undertook a descriptive epidemiology study to evaluate the presence and distribution of HPV genotypes and correlated these with cervical disease status in this population.

## Results

### Study population

At enrollment in the study, the mean age of the 301 HIV-infected women was 34 years (standard deviation ±8.9). A large majority (78.1%) were married or cohabiting with their husband. About half (50.2%) had fewer than 4 years of education (primary school or below), and under half of them (44.5%) reported a family income ≤80.5 US Dollars per month. Less than a quarter (24.3%) self-reported ever having a sexually transmitted infection (STI). A majority (62.5%) of participants reported their partners being HIV-infected. One-sixth of the participants (15.9%) reported exposure to smoking (inclusive of 2.7% current smokers, 4.7% former smokers, and 8.6% reporting second-hand smoking exposure), about two-fifths (58.8%) reported condom use with regular partners, more than two-thirds (70.8%) had 2 or fewer lifetime sexual partners, and the mean age of first sexual intercourse was 20 years (standard deviation ±3.2). The mean CD4+ cell count was 571 cells/μL, more than half (193/301, 64.1%) were taking ART, and the mean duration of being on ART was 29 months.

Cytology results revealed 252/301 (83.7%) women with ‘negative for intraepithelial lesion or malignancy’ (NILM), 33 (11.0%) with atypical squamous cells of undetermined significance (ASC-US), 11 (3.7%) with low-grade squamous intraepithelial cells (LSIL), and 4 (1.3%) with high-grade squamous intraepithelial cells (HSIL). One sample was unsatisfactory for evaluation. Colposcopic-histopathologic diagnosis revealed 251/301 (83.4%) women with no cervical intraepithelial neoplasia (CIN), 22 (7.3%) with CIN1, 16 (5.3%) with CIN2, and 12 (4%) with CIN3 lesions. The composite cytology-colposcopic-histopathological diagnosis (n = 301) showed no CIN (i.e., no CIN on colposcopy-histology and NILM on cytology) in 251/301 (83.4%) women, CIN1 (i.e., CIN1 on colposcopy-histology or ASC-US/LSIL on cytology) in 22/301 (7.3%), CIN2 (i.e., CIN2 on colposcopy-histology or HSIL on cytology) in 16/301 (5.3%), and CIN3 (on colposcopy-histology) in 12/301 (4.0%) women. Thus, CIN grade 2 or worse (i.e., CIN2+) lesions were present in 28/301 (9.3%) women.

### Distribution of HPV genotypes

Over two-fifths (131/301, 43.5%) of the HIV-infected women in the study had presence of at least one HPV genotype. A total of 21 out of the 23 HPV genotypes identifiable by the HPV genotyping kit were detected among the 131 participants in the study with HPV, including all 13 carcinogenic HPV genotypes (HPV16, 18, 31, 33, 35, 39, 45, 51, 52, 56, 58, 59 and 68) (Table 1). The five most common carcinogenic HPV genotypes were HPV52 (7.3%), HPV58 (6.6%), HPV18 (6.3%), HPV16 (6.0%), and HPV33 (5.3%). Among 131 women with any HPV infection, 83 (63.4%) were infected with a single HPV genotype, while 48 (36.6%) had multiple (≥2) genotypes. Infection with two genotypes (dual infection) accounted for almost two-thirds (32/48, 66.7%) of multiple infections, and HPV16 and HPV18 were the most frequent dual infections (3/32, 9.4%).

**Table 1:** **Prevalence of HPV genotypes stratified by composite cytology-colposcopic-histopathological CIN diagnoses**

**HPV infection groupings/types**	**Total n (%)**	**No CIN (N = 251) n (%)**	**CIN1 (N = 22) n (%)**	**CIN2+ (N = 28) n (%)**	***χ*** ^***2***^_***trend***_	***p***
Overall HPV infection	131(43.5)	93(37.1)	14(63.6)	24(85.7)	28.04	***<0.001***
Carcinogenic HPV infection						
Single carcinogenic HPV type	80(26.6)	57(22.7)	7(31.8)	16(57.1)	26.19	***<0.001***
Multiple carcinogenic HPV types	33(11.0)	21(8.4)	4(18.2)	8(28.6)	19.59	***<0.001***
Possibly carcinogenic HPV infection	15(5.0)	8(3.2)	5(22.7)	2(7.1)	4.84	***0.035*** ^***a***^
Non-carcinogenic/unknown carcinogenic HPV infection	34(11.3)	26(10.4)	6(27.3)	2(7.1)	0.12	0.768^a^
Individual HPV genotypes:						
Carcinogenic HPV genotypes						
HPV16	18(6.0)	9(3.6)	1(4.6)	8(28.6)	23.71	***<0.001*** ^***a***^
HPV18	19(6.3)	14(5.6)	1(4.6)	4(14.3)	2.46	0.121^a^
HPV31	9(3.0)	4(1.6)	2(9.1)	3(10.7)	9.70	***0.008*** ^***a***^
HPV33	16(5.3)	11(4.4)	2(9.1)	3(10.7)	2.59	0.141^a^
HPV35	10(3.3)	7(2.8)	3(13.6)	0(0.0)	0.05	1.000^a^
HPV39	1(0.3)	1(0.4)	0(0.0)	0(0.0)	0.18	1.000^a^
HPV45	2(0.7)	2(0.8)	0(0.0)	0(0.0)	0.36	0.878^a^
HPV51	2(0.7)	2(0.8)	0(0.0)	0(0.0)	0.36	0.878^a^
HPV52	22(7.3)	14(5.6)	1(4.6)	7(25.0)	11.18	***0.003*** ^***a***^
HPV56	16(5.3)	14(5.6)	2(9.1)	0(0.0)	0.80	0.433^a^
HPV58	20(6.6)	13(5.2)	2(9.1)	5(17.9)	6.56	***0.016*** ^***a***^
HPV59	6(2.0)	4(1.6)	1(4.6)	1(3.6)	0.94	0.556^a^
HPV68	16(5.3)	14(5.6)	1(4.6)	1(3.6)	0.23	0.695^a^
Possibly carcinogenic HPV genotypes						
HPV53	7(2.3)	4(1.6)	3(13.6)	0(0.0)	0.54	0.561^a^
HPV66	6(2.0)	3(1.2)	1(4.6)	2(7.1)	5.32	***0.038*** ^***a***^
HPV73	2(0.7)	1(0.4)	1(4.6)	0(0.0)	0.31	1.000^a^
HPV82	0(0.0)	0(0.0)	0(0.0)	0(0.0)	-	-
Non-carcinogenic/unknown carcinogenic HPV genotypes						
HPV6	2(0.7)	1(0.4)	1(4.6)	0(0.0)	0.31	1.000^a^
HPV11	1(0.3)	0(0.0)	1(4.6)	0(0.0)	1.45	0.166^a^
HPV42	9(3.0)	6(2.4)	2(9.1)	1(3.6)	0.84	0.442^a^
HPV43	25(8.3)	20(8.0)	4(18.2)	1(3.6)	0.03	1.000^a^
HPV44	0(0.0)	0(0.0)	0(0.0)	0(0.0)	-	-
HPV83	3(1.0)	3(1.2)	0(0.0)	0(0.0)	0.54	0.847^a^

^a^Fisher’s exact test. Statistically significant (p < 0.05) estimates are bolded and *italicized*. CIN, cervical intraepithelial neoplasia. CIN2+, cervical intraepithelial neoplasia grade 2 or worse. HPV, human papillomavirus.

A total of 113 out of 301 women (37.6%) had at least one carcinogenic HPV type. Among these, 80 women (70.8%) had a single carcinogenic HPV type infection, while 33 (29.2%) women had more than 2 carcinogenic HPV infections. Only 15/301 (5.0%) women were reported to be infected with ‘possibly-carcinogenic’ HPV types, and 34 (11.3%) women were detected with non-carcinogenic types or types of unknown carcinogenicity.

### HPV genotype prevalence by cervical diagnosis

The prevalence of HPV infection detected in all women, and women with and without cervical neoplasia is given in Table 1. The overall prevalence of HPV infection (carcinogenic, ‘possibly carcinogenic’ and non/unknown-carcinogenic HPV types) was 93/251 (37.1%) in women without CIN, 14/22 (63.6%) in CIN1, and 24/28 (87.5%) in women with CIN2+ lesions (*p*-for trend <0.001). The most common carcinogenic HPV genotypes in individual cervical disease categories were HPV18 (5.6%) in women with no CIN, HPV35 (13.6%) in CIN1, and HPV16 (28.6%) in CIN2+. The trend for increasing prevalence of HPV genotypes with increasing severity of cervical disease grades was significant for carcinogenic HPV genotype (*p* < 0.001), any single or multiple carcinogenic HPV (both for *p* < 0.001), ‘possibly carcinogenic’ HPV (*p* = .035), HPV16 (*p* < 0.001), as well as non-HPV16 carcinogenic HPV (*p* < 0.001) (Table 1).

### Trends in HPV prevalence by age, CD4 counts and ART duration

**Figure 1:**
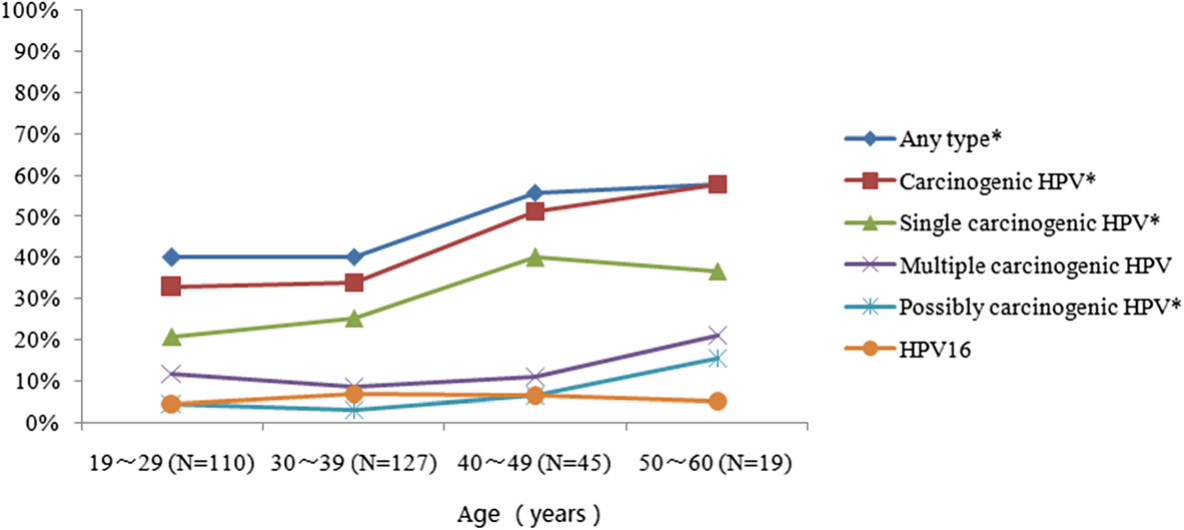
**Age-specific prevalence of HPV infection among HIV-infected women in Yunnan, China.** *p value for trend was <0.05, after adjusting for cervical lesion. HPV, Human Papillomavirus. Carcinogenic HPV includes HPV 16, 18, 31, 33, 35, 39, 45, 51, 52, 56, 58, 59 and 68. Possibly carcinogenic HPV includes HPV 53, 66, 73 and 82. With aging, *p*-value for trend for any type, carcinogenic HPV, single carcinogenic HPV and possibly carcinogenic HPV were 0.009, 0.001, 0.002 and 0.043, respectively.

The age distribution of HPV infection prevalence in the study population is depicted in Figure 1. After adjusting for severity of cervical lesions, increasing age of participants was associated with higher detection of HPV infections overall. In particular, this increase in proportion across age categories was statistically significant for the detections of any HPV, carcinogenic HPV, single carcinogenic HPV, and ‘possibly carcinogenic’ HPV genotypes (*p* = 0.009, 0.001, 0.002, and 0.043 respectively).

**Figure 2:**
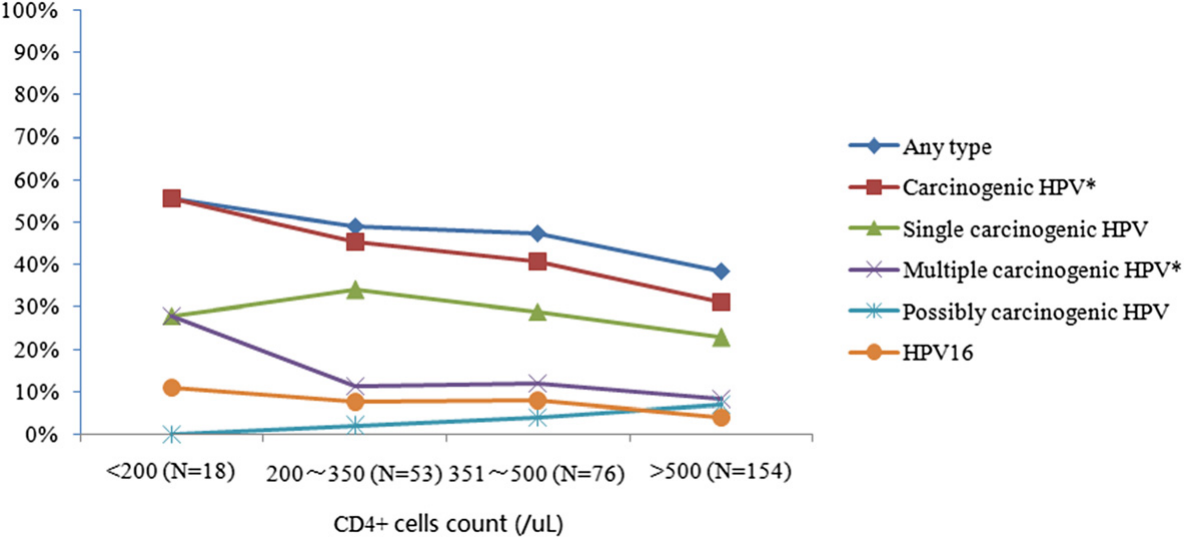
**Prevalence of HPV infection by CD4+ cells count group among HIV-infected women in Yunnan, China.** *p value for trend was <0.05, after adjusting for ART therapy status. HPV, Human papillomavirus. Carcinogenic HPV includes HPV 16, 18, 31, 33, 35, 39, 45, 51, 52, 56, 58, 59 and 68. Possibly carcinogenic HPV includes HPV 53, 66, 73 and 82. With CD4+ cell count increasing, *p*-value for trend for carcinogenic HPV and multiple carcinogenic HPV were 0.010 and 0.020, respectively.

The relative proportion of HPV infection by CD4+ cell count categories, after adjusting for ART status, is graphically displayed in Figure 2. In general, lower CD4+ cell counts were associated with higher detection of HPV infections. However, the proportions varied significantly (*p* < 0.05) only for the detections of carcinogenic HPV and multiple carcinogenic HPV genotypes.

**Figure 3:**
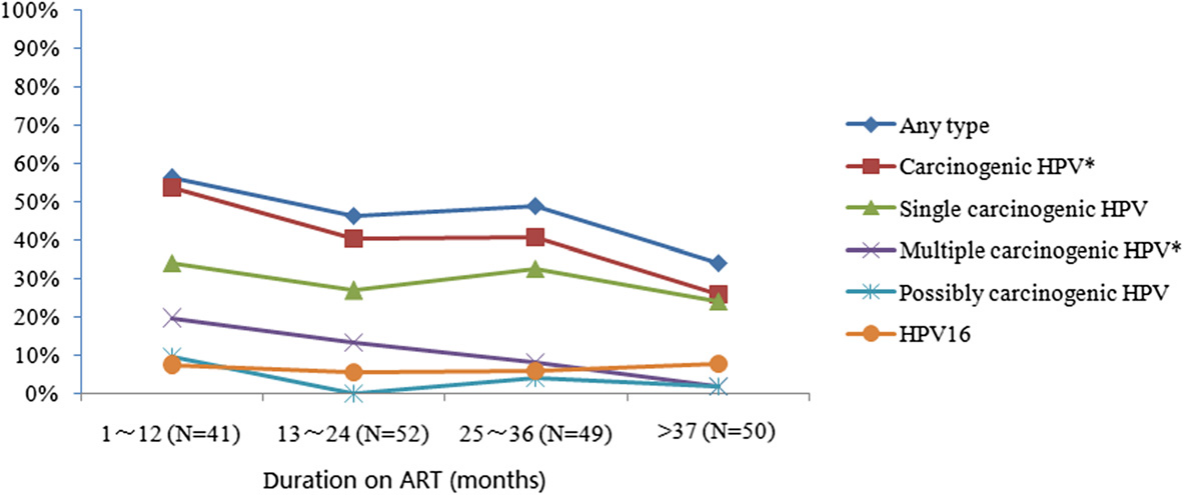
**Prevalence of HPV infection by duration of receiving ART group among HIV-infected women in Yunnan, China.** *p value for trend of univariate analysis was <0.05. HPV, Human Papillomavirus. Carcinogenic HPV includes HPV 16, 18, 31, 33, 35, 39, 45, 51, 52, 56, 58, 59 and 68. Possibly carcinogenic HPV includes HPV 53, 66, 73 and 82. ART, Antiretroviral Therapy. With increasing duration on ART, *p*-value for trend for carcinogenic HPV and multiple carcinogenic HPV were 0.013 and 0.002, respectively.

Figure 3 presents HPV genotype groupings by duration (in months) on being on ART. Longer duration of being on ART was associated with lower detection of HPV infections. Statistically significant (*p* < 0.05) lower HPV detections associated with categories of longer duration on ART were observed for the groupings of carcinogenic HPV and multiple carcinogenic HPV genotypes.

### Risk factors of HPV infection

Table 2 reports the unadjusted and the multivariable-adjusted associations of single and multiple carcinogenic HPV infections with sociodemographic, behavioral, and HIV-related covariates. In the unadjusted analysis, having single carcinogenic HPV infection was directly associated with age and having a cervical lesion (≥CIN1), and inversely associated with CD4+ cell counts (Table 2) while having multiple carcinogenic HPV infection was directly associated only with having a cervical lesion (≥CIN1). In the multivariable-adjusted multinomial logistic regression analysis, presence of single carcinogenic HPV infection was independently associated with both increasing age (adjusted odds ratio [AOR]: 1.04, 95% confidence interval [CI]: 1.01-1.07, *p* = 0.012) and having a cervical lesion (≥CIN1) (AOR: 5.37, 95% CI: 2.57-11.23, *p* < 0.001) while presence of multiple carcinogenic HPV infections were independently associated with having a cervical lesion (≥CIN1) (AOR: 7.39, 95% CI: 2.98-18.29, *p* < 0.001).

**Table 2:** **Risk factors of single and multiple carcinogenic HPV infection in unadjusted and multivariable-adjusted multinomial logistic regression**

**Factor**	**Total**	**No carcinogenic** **HPV infection** ^**a**^	**Single carcinogenic HPV**			**Multiple carcinogenic HPV**		
	**N (%)**	**n (%)**	**n (%)**	**Unadjusted OR (95%CI)**	***p***	**n (%)**	**Unadjusted OR (95%CI)**	***p***
**Unadjusted analysis**								
Age								
(per year increase)	301(100.0)	188(100.0)	80(100.0)	1.03(1.00-1.06)	***0.025***	33(100.0)	1.02(0.98-1.01)	0.279
Married status								
No-cohabiting	66(21.9)	39(20.7)	17(21.3)	1.00		10(30.3)	1.00	
Married/cohabiting	235(78.1)	149(79.3)	63(78.8)	0.97(0.51-1.84)	0.926	23(69.7)	0.60(0.27-1.37)	0.226
Completed years of education								
≤4	151(50.2)	98(52.1)	38(47.5)	1.00		15(45.5)	1.00	
≥5	150(49.8)	90(47.9)	42(52.5)	1.20(0.71-2.03)	0.488	18(54.5)	1.31(0.62-2.75)	0.480
Family income (USD)								
≤80.5 per month	134(44.5)	79(42.0)	37(46.3)	1.00		18(54.5)	1.00	
>80.5 per month	167(55.5)	109(58.0)	43(53.8)	0.84(0.50-1.43)	0.523	15(45.5)	0.60(0.29-1.27)	0.184
Smoking								
No	253(84.1)	157(83.5)	70(87.5)	1.00		26(78.8)	1.00	
Yes	48(15.9)	31(16.5)	10(12.5)	0.72(0.34-1.56)	0.408	7(21.2)	1.36(0.54-3.42)	0.509
Patient’s partner infected with HIV								
No	113(37.5)	70(37.2)	34(42.5)	1.00		9(27.3)	1.00	
Yes	188(62.5)	118(62.8)	46(57.5)	0.80(0.47-1.37)	0.419	24(72.7)	1.58(0.70-3.60)	0.274
Age at first sex								
≤18 years	95(31.6)	62(33.0)	24(30.0)	1.00		9(27.3)	1.00	
>18 years	206(68.4)	126(67.0)	56(70.0)	1.15(0.65-2.02)	0.633	24(72.7)	1.31(0.58-2.99)	0.518
Lifetime sex partners								
≤2	213(70.8)	137(72.9)	55(68.8)	1.00		21(63.6)	1.00	
>2	88(29.2)	51(27.1)	25(31.3)	1.22(0.69-2.61)	0.494	12(36.4)	1.54(0.80-3.34)	0.281
History of STI								
No	228(75.7)	136(72.3)	66(82.5)	1.00		26(78.8)	1.00	
Yes	73(24.3)	52(27.7)	14(17.5)	0.56(0.29-1.07)	0.080	7(21.2)	0.70(0.29-1.72)	0.442
Condom use								
No	124(41.2)	84(44.7)	26(32.5)	1.00		14(42.4)	1.00	
Yes	177(58.8)	104(55.3)	54(67.5)	1.68(0.97-2.91)	0.065	19(57.6)	1.10(0.52-2.32)	0.810
Total number of pregnancies								
<3	160(53.2)	101(53.7)	41(51.3)	1.00		18(54.5)	1.00	
≥3	141(46.8)	87(46.3)	39(48.8)	1.10(0.65-1.87)	0.710	15(45.5)	0.97(0.46-2.03)	0.930
CD4+ cell count								
(increase by 100/μL)	301(100.0)	188(100.0)	80(100.0)	0.89(0.80-0.99)	***0.039***	33(100.0)	0.91(0.78-1.06)	0.230
ART therapy								
No	108(35.9)	71(37.8)	24(30.0)	1.00		13(39.4)	1.00	
Yes	193(64.1)	117(62.2)	56(70.0)	1.42(0.81-2.48)	0.225	20(60.6)	0.93(0.44-1.99)	0.859
Duration on ART (years)								
No	108(35.9)	71(37.8)	24(30.0)	1.00		13(39.4)	1.00	
<2	94(31.2)	51(27.1)	28(35.0)	1.62(0.85-3.12)	0.146	15(45.5)	1.61(0.70-3.67)	0.260
≥2	99(32.9)	66(35.1)	28(35.0)	1.26(0.66-2.38)	0.487	5(15.2)	0.41(0.14-1.22)	0.111
Cervical lesion								
No	251(83.4)	173(92.0)	57(71.3)	1.00		21(63.6)	1.00	
Yes	50(16.6)	15(8.0)	23(28.8)	4.65(2.27-9.52)	***<0.001***	12(36.4)	6.59(2.72-15.95)	***<0.001***
	**Total**	**No carcinogenic HPV infection** ^**a**^	**Single carcinogenic HPV**			**Multiple carcinogenic HPV**		
	**N (%)**	**n (%)**	**n (%)**	**Adjusted OR (95%CI)**	***p***	**n (%)**	**Adjusted OR (95%CI)**	***p***
**Multivariable-adjusted analysis** ^**b**^								
Age								
(per year increase)	301(100.0)	188(100.0)	80(100.0)	1.04(1.01-1.07)	***0.012***	33(100.0)	1.03(0.99-1.08)	0.132
CD4+								
(increase by 100 μL)	301(100.0)	188(100.0)	80(100.0)	1.00(1.00-1.00)	0.143	33(100.0)	1.00(1.00-1.00)	0.422
Cervical lesion								
No	251(83.4)	173(92.0)	57(71.3)	1.00		21(63.6)	1.00	
Yes	50(16.6)	15(8.0)	23(28.8)	5.37(2.57-11.23)	***<0.001***	12(36.4)	7.39(2.98-18.29)	***<0.001***

Statistically significant (p < 0.05) estimates are bolded and *italicized*. OR: Odds Ratio. 95%CI: Lower limits and upper limits of the 95% Confidence intervals. CIN, cervical intraepithelial neoplasia. HPV, human papillomavirus. STI, sexually transmitted infection. ART, antiretroviral therapy.

Carcinogenic HPV includes HPV 16, 18, 31, 33, 35, 39, 45, 51, 52, 56, 58, 59 and 68.

^a^No carcinogenic HPV infection refers to absence of all 13 types of carcinogenic HPV.

^b^Only factors that were statistically significant (*p* < 0.05) in the unadjusted analysis were included in the multivariable-adjusted analysis.

## Discussion

Persistent infection with carcinogenic HPV is a necessary, though not sufficient, cause for invasive cervical cancer. HIV-infected women are more susceptible to HPV than HIV-uninfected women. Within China, the HIV/AIDS epidemic is diverse, with some of the highest rates in southwest China, especially in Yunnan province [11].

The current study is the first study from China to provide a comprehensive assessment of the distribution of HPV genotypes among HIV-infected women in Yunnan using rigorously-ascertained cervical disease diagnoses. To avoid unnecessary invasive diagnosis procedures and increase the accuracy of CIN diagnosis than relying on any one approach (cytology or colposcopy-histology) alone, our study combined both cytology results and colposcopy-histologic diagnosis to compute composite disease endpoints. Thus we maximized the detection of cervical lesions, limited any selection bias, and increased the representativeness of our findings. The results from our cohort are therefore representative of HIV-infected women who seek cervical cancer screening services in Yunnan and our findings could add to the national and global data of HPV distribution among HIV-infected populations.

Overall, at least one HPV genotype was detected in over two-fifths of women and at least one carcinogenic HPV genotype was present in over one-third of HIV-infected women. The prevalence of any HPV infection (43.5%) was almost three times higher than the corresponding rate among the general (HIV-uninfected) population in China [12], and the rate of carcinogenic HPV detection (37.5%) was more than twice as high as corresponding rates reported in studies conducted in the general Chinese population [12]. These detection rates among HIV-infected women in our study are comparable to that reported from two other Chinese studies among HIV-infected women [9,10]; yet these studies lacked the detailed cervical disease assessment accomplished in our study.

The prevalence of HPV infection has ranged widely in studies among HIV-infected women in Asia [6,8,13-18]. A global meta-analysis in 2006 reviewed 20 contemporaneous studies and demonstrated the overall prevalence of HPV infection to be 36.3% among women without cervical abnormalities, and increased with increased severity of cervical disease [6]. Our cohort noted 37.1% of women without CIN lesion were infected any HPV, and carcinogenic HPV prevalence rate (31.1%) was similar to that reported from Asia (31.1%), Europe (32.4%), North America (31.4%), but lower than that from Africa (56.6%) and South/Central America (57.3%) [6].

Consistent with meta-analysis of HPV types distribution among HIV-infected women [6], HPV16 was not the most common type in the overall population in our study. Some experts have hypothesized that HPV16 has better evolutionary ability to escape the effects of immune surveillance, while non-HPV16 genotypes are often better controlled by immune response, such as among women with competent immune systems (e.g., HIV-infected women with CD4 > 500/μL) [19,20]. In women with progressively weakening immune systems (i.e., with declining CD4+ cell counts), it is thought that the immune control over non-HPV16 types is lost and they appear with greater preponderance than in women with competent immune systems [19,20]. Indeed, we did observe an increase of non-HPV16 carcinogenic HPV genotypes with progressively worsening immune status reflected by lower CD4+ count categories (*p*-for trend = 0.029) and shorter duration of ART (*p*-for trend = 0.004), consistent with the hypothesized loss of immune control over non-HPV16 types. However, HPV16 was still the most commonly detected carcinogenic type among women with cervical disease. Also, apart from non-HPV16 carcinogenic HPV types, we found significant differences regarding ART duration and CD4+ counts in any HPV and any carcinogenic HPV (all *p*-for trend < 0.05). Our results point to the need for further exploration of differences in immune control between HPV16 and non-HPV16 carcinogenic genotypes, and evaluating these associations with cervical cancer risk. In addition, it may be important to study the effect of early ART initiation on reduction in risk of carcinogenic HPV-induced neoplastic lesions, and if it differs by HPV genotypes involved. It will also be important to study the etiologic significance of concurrently present non/unknown-risk genotypes, some of which such as HPV43 had higher prevalence than even other carcinogenic HPV genotypes.

We noted that 29.2% of carcinogenic HPV positive women were infected with multiple carcinogenic HPV genotypes. This is similar to the rate of multiple HPV infections (32.2%) among carcinogenic HPV positive women in the general population when using GP5+/GP6+ polymerase chain reaction (PCR) assay as the HPV detection method [21]. The proportion of multiple carcinogenic HPV types was even higher (50%, 8/16) among women with CIN2 or worse lesion. This high rate of multiple HPV infections (consistent with the rates of 12%-87.5% in previous studies in HIV-infected populations), confounds the measurement of genotype-specific fractions attributable to individual cervical disease categories [13,15,22]. A higher prevalence of multiple HPV types may be due to different reasons, including higher persistence and lower clearance of HPV in the setting of HIV infection, continued sexual exposure to novel HPV types, reactivation of latent HPV during periods of severe immune-suppression, or may be due to high relative analytical sensitivity of the PCR assay.

It was interesting to note that age was a significant factor only with the presence of single carcinogenic infection but not multiple carcinogenic infections in the multivariable-adjusted analysis. While this lack of statistical association of age and multiple carcinogenic infections is intriguing, it likely reflects the underpowered nature of the study for evaluating this outcome, since we only had 33 cases of multiple carcinogenic infections overall and only 8 among women with CIN2+ disease. Indeed, we did not find any significant differences in multiple carcinogenic HPV infections relating to lower CD4+ cell counts, sexual behaviors, or ART therapy. (Presence of cervical lesions was a significant factor for both single and multiple carcinogenic infection outcomes, as expected, since HPV is a causative agent of cervical neoplastic lesions).

We noted that there was no clear peak of carcinogenic HPV prevalence in young (<30 years) women, unlike age-specific HPV prevalence surveys in China and globally [23,24]. There were no specific occupational or HIV-related risk factor differences or characteristics that distinguished this cohort from other HIV-infected women in Yunnan or China. Whether the atypical age-specific curves are reflective of setting-specific cultural factors such as lower prevalence of premarital or extramarital sexual relations needs further exploration.

The currently available prophylactic HPV vaccines offer protection against HPV16 and HPV18 and some cross-protection to a few associated genotypes, and the future nonavalent vaccine could extend the protection against HPV 31/33/45/52/58. Although the vaccines have been shown to be safe and immunogenic, there are no studies yet on the efficacy of these vaccines among HIV-infected women [25]. Hence, there is still no unequivocal recommendation to vaccinate all HIV-infected women. In any case, given the high rates of HPV infection, a vast majority of HIV-infected women may already be infected with types covered by the vaccine. Therefore, screening and early detection of precancerous lesions will continue to be important for HIV-infected women, particularly as they live longer lives due to ART, and remain at continued risk for development and progression of HPV-induced cervical lesions.

The strengths of our study included the choice of combined cytological and colposcopy-histological diagnosis, to compute the final disease categorization for women. This allowed for better disease ascertainment than relying on the results of cytology or colposcopy-histology alone. Our study also had several limitations. Firstly, subtle immunosuppression could not be measured by the enumeration of circulating CD4+ cells. Secondly, this study was cross-sectional, so results only could show an association but no evidence of causality. Thirdly, some behavioral factors such as drug use and commercial sex work were not captured in the questionnaire of our study, both of which are known to be significant risk factors for HIV-infection for many women in China, particularly in Yunnan. Finally, we note the absence of data on plasma HIV RNA data, nadir CD4 count, and duration of HIV infection while analyzing risk factors as a limitation of the study.

## Conclusions

In summary, in this cross-sectional study, we evaluated the diverse distribution of HPV genotypes and factors associated with HPV infection among HIV-infected women in Yunnan province. These results are similar to those reported among HIV-infected women in countries with similar levels of the HIV epidemic. The diversity of HPV genotypes in this population emphasizes the need for broad spectrum (polyvalent) HPV vaccines for eventual primary prevention, as well as the immediate necessity of regular and vigilant screening for cervical disease in this population.

## Methods

### Study population

The study was conducted in a HIV-clinic in the Mangshi prefecture of Yunnan province, where HIV-infected women have been provided with free cervical cancer screening services since March 2009. Study subjects were recruited based on linkages with HIV voluntary counseling and testing (VCT) centers and referrals of known HIV-infected women from the provincial and prefecture-level Center for Disease Control (CDC)-affiliated ART clinics. The program staff, in close linkages with the CDC staff and the federally-funded “*Four Frees and One Care*” program that provided ART for HIV-infected women, was responsible for recruitment and retention of participants. For our study, we selected a cohort of HIV-infected women based on the following eligibility criteria: (i) 18 years of age or older (for informed consent); (ii) not currently pregnant; (iii) no history of prior treatment for cancer of the cervix; (iv) no history of hysterectomy; (v) no prior history of cervical cancer screening; and (vi) mentally and physically fit for undergo a pelvic examination. Participants were recruited regardless of CD4+ cell counts or current status of receiving ART. The study protocol was approved by the Institutional Review Boards at Cancer Institute and hospital, Chinese Academy of Medical Sciences (CICAMS) and Vanderbilt University. All participants gave written, informed consent.

### Study procedures

After detailed explanation of study procedures, written informed consent was obtained. A structured questionnaire was used to collect socio-demographic information and key bio-behavioral risk factors relevant to HIV/AIDS or cervical cancer. All enrolled participants underwent a complete external physical examination and venous blood collection for CD4+ T-lymphocyte count. A pelvic examination was performed with sample collection by Ayre’s spatula and endocervical cytobrush (for HPV testing and liquid-based cervical cytology). All participants underwent visual screening and diagnostic colposcopy by trained gynecologist. Colposcopically-directed cervical biopsy, endocervical curettage (ECC), and loop electrosurgical excision procedures (LEEP) were advised and performed on consenting participants with clinical evidence of cervical abnormalities.

Biopsy specimens were processed and interpreted by consulting pathologists affiliated with the Yunnan Cancer Hospital. Colposcopy and histology results were reported as per the Richart cervical intraepithelial neoplasia [CIN] grading system [26] and cervical cytology results were reported as per revised (2001) Bethesda classification [27]. The final diagnosis for each woman was based on combining the results of the worst diagnosis on both colposcopy/histology and cytology: ‘No CIN’ (normal colposcopy/histology and cytology results of ‘negative for intraepithelial lesions of malignancy’ [NILM]), ‘CIN1’ (CIN1 lesions on colposcopy/histology or cervical cytology results of either atypical squamous cells of undetermined significance [ASC-US] or low-grade squamous intraepithelial cells [LSIL], ‘CIN2+’ (CIN2 or CIN3 on colposcopy/histology or high-grade squamous intraepithelial cells [HSIL] on cervical cytology).

We performed HPV genotyping on cervical specimens using polymerase chain reaction (PCR)-based amplification and commercial DNA-chip technology kit (Yaneng Bioscience (Shenzhen) Co. Ltd, P20120401), an SFDA-approved assay that has been extensively used in the past [28]. The kit could amplify and detect the presence of 23 HPV types: 6, 11, 16, 18, 31, 33, 35, 39, 42, 43, 44, 45, 51, 53, 52, 56, 58, 59, 66, 68, 73, 82, and 83. The threshold for detection of HPV was 1.0 × 10^3^ viral copies/μL. According to Bouvard classification [2], HPV genotypes groups were carcinogenic HPV (including HPV16, 18, 31, 33, 35, 39, 45, 51, 52, 56, 58, 59 and 68), ‘possibly carcinogenic’ HPV (including HPV53, 66, 73 and 82) and non-carcinogenic HPV/HPV of unknown carcinogenicity (including HPV6, 11, 42, 43, 44 and 83).

### Statistical analyses

The statistical analysis was conducted using IBM SPSS 17.0. The distribution of HPV infection and genotypes were presented as proportion. Chi-square tests were used for assessing the relationship between the distribution of HPV genotypes and different grades of cervical disease. Unadjusted and multivariable-adjusted multinomial logistic regression were used to assess the effect of socio-demographic, sexual, reproductive and HIV-infection related characteristics on the presence of single and multiple types of carcinogenic HPV, and adjusted odds ratios (AOR) and 95% confidence intervals (95%CI) of factors independently associated with single and multiple carcinogenic HPV infection among HIV-infected women were calculated. A two-tailed p-value of <0.05 was considered significant.

### Ethics statement

The study protocol was approved by the institutional ethics committees of the collaborating institutions and has therefore been performed in accordance with the ethical standards laid down in the 1964 Declaration of Helsinki and its later amendments. All study participants gave written, informed consent.

## Abbreviations

HPV: Human papillomavirus
HIV: Human immunodeficiency virus
ART: Antiretroviral therapy
STI: Sexually transmitted infection
CIN: Cervical intraepithelial neoplasia
CIN2+: cervical intraepithelial neoplasia grade 2 or worse
ASC-US: Atypical squamous cells of undetermined significance
LSIL: Low-grade squamous intraepithelial cells
HSIL: High-grade squamous intraepithelial cells
